# Active Giant Cell Vasculitis Diagnosis with ^68^Ga PSMA PET/CT Imaging

**DOI:** 10.4274/mirt.galenos.2019.90532

**Published:** 2021-02-09

**Authors:** Muhammet Sait Sağer, Seçkin Bilgiç, Lebriz Uslu, Sertaç Asa, Güneş Sağer, Kerim Sönmezoğlu

**Affiliations:** 1 İstanbul University-Cerrahpaşa, Cerrahpaşa Faculty of Medicine, Department of Nuclear Medicine, İstanbul, Turkey; 2 Kartal Dr. Lütfi Kırdar City Hospital, Clinic of Pediatry, İstanbul, Turkey

**Keywords:** Vasculitis, GCA, PSMA PET, 18F-FDG PET

## Abstract

Vasculitis is a multisystem disease characterized by inflammation with infiltration of leukocytes into the blood vessels. Giant cell arteritis (GCA) is the most common form of vasculitis that mostly affects medium- and large-sized arteries. ^18^Fluorine-fluorodeoxyglucose (^18^F-FDG) positron emission tomography/computed tomography (PET/CT) is increasingly used to diagnose inflammation of large arteries in GCA. Galium-68 prostate-specific membrane antigen (PSMA) PET/CT has a vital role in the assessment of patients with prostate cancer for recurrence and metastasis of the disease. Various benign and non-prostate malignant conditions may give rise to increased PSMA uptake. Herein, we demonstrate that PSMA uptake can be seen in GCA.

## Abstract (tr)

Vaskülit, lökositlerin kan damarlarına infiltrasyonu sonucunda iltihaplanma ile karakterize multisistemik bir hastalıktır. Dev hücreli arterit (DHA), çoğunlukla orta büyüklükte ve büyük arterleri etkileyen en yaygın vaskülit şeklidir. ^18^Flor-florodeoksiglukoz (^18^F-FDG) pozitron emisyon tomografi/bilgisayarlı tomografi (PET/BT), DHA tanısında yaygın olarak kullanılır. Galyum-68 prostat spesifik membran antijeni (PSMA) PET/BT, nüks ve metastatik hastalığı olan prostat kanseri hastalarının değerlendirilmesinde önemli bir role sahiptir. PSMA tutulumuna neden olabilecek çeşitli iyi huylu ve prostat dışı malign durumlar görülebilmektedir. Bu olgu ile büyük damar vaskülitinde PSMA tutulumunu görülebileceğini göstermek istedik.

## Figures and Tables

**Figure 1:**
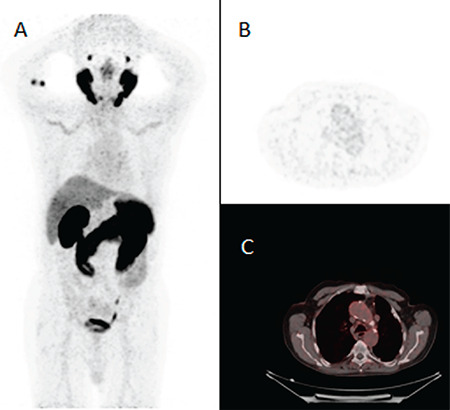
A-56-year-old male patient diagnosed with prostate cancer was referred to nuclear medicine for gallium-68 (^68^Ga) prostate-specific membrane antigen (PSMA) positron emission tomography/computed tomography (PET/CT) imaging. An intravenous solution of 4 mCi ^68^Ga PSMA was administered followed by whole-body PET/CT imaging at 1 hour post administration of intravenous solution. No recurrence or metastatic PSMA uptake was observed for prostate cancer. However, increased PSMA uptake was noted bilaterally in the subclavian arteries and common carotid arteries in maximum intensity projection (A), axial PET (B), and axial fusion (C) images. PSMA is a type 2 transmembrane protein with high expression in prostate carcinoma cells (1). ^68^Ga PSMA PET/CT has an important role in the assessment of patients with prostate cancer and recurrence and metastasis of the disease (2). ^68^Ga PSMA uptake has been evident in various solid malignant neoplasms such as neuroendocrine tumors, renal cell carcinoma, breast cancer, and differentiated thyroid cancer (3). This form of vessel uptake can be seen with ^18^fluorine-fluorodeoxyglucose (^18^F-FDG) PET in vasculitis. This patient was diagnosed with giant cell vasculitis. Recognition of the potential sources of false-positive and false-negative findings is important for accurate interpretation of PSMA-targeted PET imaging studies.

**Figure 2:**
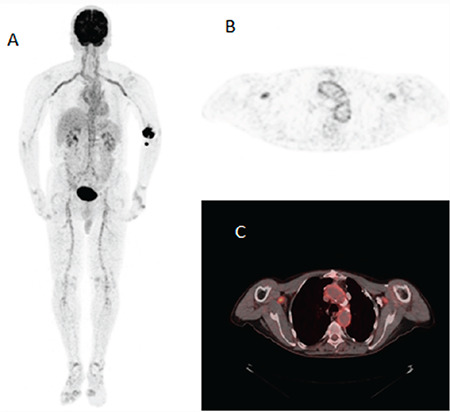
^18^F-FDG PET/CT images showed bilateral increased ^18^F-FDG uptake in the subclavian arteries and common carotid arteries in the maximum intensity projection (A) axial PET (B), and axial fusion (C) images. ^18^F-FDG uptake was higher than that of PSMA. Giant cell arteritis (GCA), also called temporal arteritis, is a granulomatous inflammation of the aorta and its main branches, most often occurring in patients aged >50 years (4). Vasculitis can be distributed locally in the branches of the internal and external carotid arteries or the aorta. Visual vascular uptake higher than that of liver resulted in the highest diagnostic accuracy for the detection of GCA (5). ^18^F-FDG PET/CT is routinely used for the diagnosis of vasculitis and evaluation of treatment response.
